# Application of Metal Oxide Nanoparticles in the Field of Potentiometric Sensors: A Review

**DOI:** 10.3390/membranes13110876

**Published:** 2023-11-07

**Authors:** Nikola Lenar, Robert Piech, Cecylia Wardak, Beata Paczosa-Bator

**Affiliations:** 1 Faculty of Materials Science and Ceramics, AGH University of Krakow, Mickiewicza 30, PL-30059 Krakow, Poland; nlenar@agh.edu.pl (N.L.);; 2 Department of Analytical Chemistry, Institute of Chemical Sciences, Faculty of Chemistry, Maria Curie-Sklodowska University, Maria Curie-Sklodowska Square 3, PL-20031 Lublin, Poland; cecylia.wardak@mail.umcs.pl

**Keywords:** potentiometric sensors, metal oxides, constructions of sensors, solid-contact electrodes, glass electrode, screen-printed electrodes, paste electrodes

## Abstract

Recently, there has been rapid development of electrochemical sensors, and there have been numerous reports in the literature that describe new constructions with improved performance parameters. Undoubtedly, this is due to the fact that those sensors are characterized by very good analytical parameters, and at the same time, they are cheap and easy to use, which distinguishes them from other analytical tools. One of the trends observed in their development is the search for new functional materials. This review focuses on potentiometric sensors designed with the use of various metal oxides. Metal oxides, because of their remarkable properties including high electrical capacity and mixed ion-electron conductivity, have found applications as both sensing layers (e.g., of screen-printing pH sensors) or solid-contact layers and paste components in solid-contact and paste-ion-selective electrodes. All the mentioned applications of metal oxides are described in the scope of the paper. This paper presents a survey on the use of metal oxides in the field of the potentiometry method as both single-component layers and as a component of hybrid materials. Metal oxides are allowed to obtain potentiometric sensors of all-solid-state construction characterized by remarkable analytical parameters. These new types of sensors exhibit properties that are competitive with those of the commonly used conventional electrodes. Different construction solutions and various metal oxides were compared in the scope of this review based on their analytical parameters.

## 1. Introduction

Nowadays, due to the dynamic socioeconomic development, greater attention is being paid to the protection of the environment and human health. At the same time, the requirements for analytical methods used to assess patient health, environmental conditions, and quality of products are increasing [1]. There is a constant search for methods characterized by a short waiting time for the analysis result, accuracy, and reliability, while at the same time generating low costs of performing a single analysis [2,3]. An analytical technique that meets these requirements is potentiometry, within which a group of potentiometric sensors has been developed. In the last thirty years, there has been rapid advancement of this group of electrodes, and there have been many reports in the literature that describe new constructions with improved performance parameters [4,5,6,7,8,9]. Undoubtedly, this is due to the fact that potentiometric sensors are characterized by very good analytical parameters, and at the same time, they are cheap and easy to use, which distinguishes them from other analytical tools. One of the observed trends in the development of potentiometric sensors is the search for new functional materials and the miniaturization of electrodes.

The constant search for new materials and construction solutions for potentiometric sensors emerges from the need for reliable analytical tools characterized by low costs, rapid responses, and miniature size. Conventional electrodes with an inner solution including the commonly known glass electrode (for pH measurements) exhibit satisfying analytical parameters; however, their construction results in a number of limitations. Thanks to years of research, conventional electrodes can now be replaced by all-solid-state sensors such as solid-contact electrodes, paste electrodes, or screen-printed electrodes, which are characterized by both great analytical and performance parameters.

Recently, there has been rapid development of all-solid-state potentiometric sensors, and there have been a lot of publications describing innovative designs with improved performance. This research focuses on achievements in the field of potentiometric sensors because of the use of metal oxides.

Metal oxides of nanostructured morphology are of both scientific and technical importance and have found application in numerous scientific disciplines, e.g., biomedicine, optics, electronics and electrochemical methods [10,11]. With a high surface-to-volume ratio, nanostructured metal oxides have been found to enhance the sensitivity, selectivity, catalytic activity, and electrical capacity of electrochemical devices. Metal oxides have been applied to date in electrochemical sensors either as a sensing material [12] or as a solid-contact layer (or paste) under the sensing membrane [13]. Electrochemical sensors can be used in association with various electrochemical detection methods, and these include conductometric, chronopotentiometric, voltammetric, amperometric, impedimetric, coulometric, and potentiometric measurements [14].

## 2. Potentiometric Sensors

With reference to the entire group of electrochemical sensors, they can be characterized as analytical tools with a small size and low purchase and maintenance cost, which account for their remarkable practicality and the possibility of performing analyses in field conditions. The use of sensors does not require high energy consumption and special laboratory conditions [8,15]. The aforementioned features of the sensors have contributed to their rapid development over the past few years.

The principle of electrochemical sensors is based on the interaction between “electricity” and “chemistry.” The part of the sensor in which “chemistry” plays the key role is the receptor part, which is responsible for the specific interaction of the analyzed ions with the sensor. The other part of the sensor converts the received chemical signal into an electrical signal (usually in the form of a current or potential change) [4]. One of the largest and most used groups of electrochemical sensors is potentiometric sensors.

**Figure 1:**
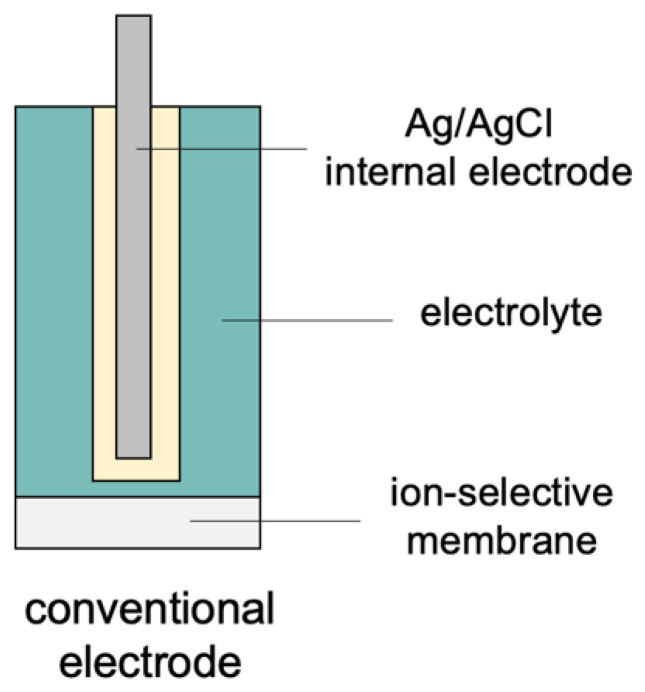
The schematic representation of the conventional electrode.

Potentiometric sensors have adopted various designs over the years and have been used for the determination of ionic analytes in samples of varying matrix and origin [13,16]. The first ion-selective electrode described in the literature was a conventional design electrode, that is, one characterized by the presence of an internal solution and an ion-exchange membrane [17]. An example of a sensor with a design of this type is a glass electrode containing an electrode that generates a membrane potential and acts as an ion-to-electron conductor (which is an Ag/AgCl semi-cell) along with an internal solution of fixed composition and a glass membrane [18,19,20,21]. Conventional electrodes with an inner solution including the commonly known glass electrode (for pH measurements) exhibit satisfying analytical parameters; however, their construction results in a number of limitations, and the main one is their fragility, which makes the electrode vulnerable to any mechanical damage and large size, which preclude the analysis of small volume samples. Conventional electrodes are also associated with the disadvantages caused by the presence of an inner solution. This construction of an electrode requires a vertical position, a stable temperature, and pressure to avoid the phase change of the inner solution. Figure 1 presents a schematic representation of the construction of the conventional electrode.

A milestone in the development of sensors was the elimination of the internal electrolyte from the electrode construction, resulting in the establishment of a new broad group of potentiometric sensors [22].

A characteristic element of the design of potentiometric sensors is the sensing layer, which is an ion-selective membrane or, in the case of screen-printed electrodes, the sensing material layer. At the sensor–solution interface, a potential difference arises. In ion-selective electrodes with a membrane, the potential depends mainly on the activity of the potential-forming ion located in the membrane and in the solution, passing from one phase to the other. In a potentiometric sensor without the membrane, where the sensing functional material is responsible for the recognition of analyzed ions, the potential arises because the redox reaction occurs near the surface of the material. The potential is created due to the exchange reactions taking place between the sensor and the analyzed solution [4,7,8,17].

**Figure 2:**
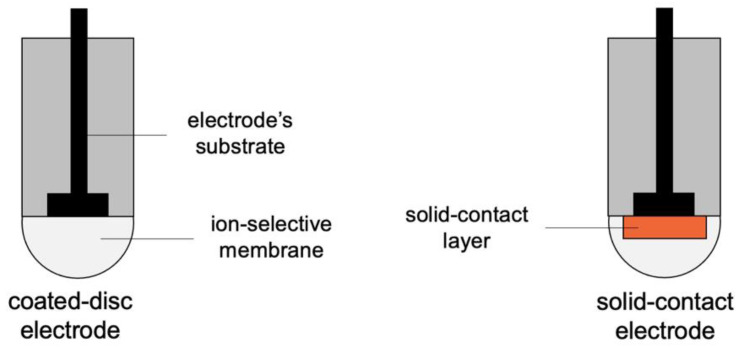
The schematic representation of the coated disc and solid-contact electrode.

The first construction of an electrode without the inner solution was presented in the literature in the early 1970s by Cattrall et al. [23]. This construction was obtained by indirectly casting the ion-selective membrane onto the surface of the electrode (as presented in Figure 2). The construction is much simpler compared to that of the conventional electrode and more resistant to mechanical damage. However, the analytical parameters of this type of sensor are noticeably worse—the potential stability is poor, and the time of response is prolonged in contrast to the electrode with the inner solution. It turned out that in order to ensure efficient ion-to-electron exchange properties, the presence of the “key” between the ion-selective membrane and the electrode is crucial.

This led to the introduction of a new group of sensors, the solid-contact electrodes, characterized by the presence of the solid-contact layer in between the membrane and the electrode. A schematic representation of the construction of the solid-contact electrode is presented in Figure 2. The presence of material that exhibits both ionic and electronic transduction properties allows the charge between the sensor and the solution to be transferred efficiently.

Materials used as solid-contact layers can be divided into materials with a high redox capacity and materials with a high double-layer capacity [7,9]. The high redox capacity is related to the electronic and ionic conductivity of materials in which exchange processes occur because of reversible redox reactions. The first group of materials implemented into the sensor was the group of conducting polymers introduced in the literature in the early 1990s by Cadogan et al., who presented the use of poly(pyrrole) (PPy) [24]. When talking about conducting polymers, the following polymers should also be mentioned: poly(3-octylthiophene) (POT) [25], poly(aniline) (PANI) [26], poly(3,4-ethylene-1,4-dioxythiophene) (PEDOT) [27], and PEDOT doped with other ions: PEDOT(PSS) and PEDOT(Cl) [27]. The group of materials with high redox capacity also includes lipophilic silver complexes [28], ferrocene [29], Prussian blue [30], Co(II)/Co(III) cobalt salts [31], molecular organic compounds such as 7,7,8,8-tetracyanoquinodimethane (TCNQ) [32] and tetrathiafulvalene (TTF) and their salts [33]. It is also worth mentioning the composite materials included in this group, such as the TTF-TCNQ material [34] or self-stabilizing monolayers based on fullerenes and TTF [35].

The second group of materials is represented by materials with a high surface area resulting from the nanometric size of particles or pores in the material structure. This group includes carbon nanomaterials (carbon nanotubes [36], carbon nanohorns [37,38], graphene [39], carbon black [40], three-dimensionally ordered macroporous (3DOM) carbon [41], fullerenes [42], mesoporous carbon colloidal CIM [43]) and metal nanoparticles—gold nanoparticles [44], platinum [45], and nanoporous gold films [46]. By combining the materials mentioned above, composite materials were obtained, such as platinum nanoparticles stabilized on PtNPs-CB carbon black [45] or PtNPs-GR graphene [47] or carbon nanotubes with bimetallic gold and copper particles MWCNTs-AuCuNPs [48]. From the point of view of the presented division, metal oxides can be classified into both groups of materials. They are described both as materials with a high redox capacity and a high double-layer capacity because they exhibit redox sensitivity and are characterized by a large surface area [49].

**Figure 3:**
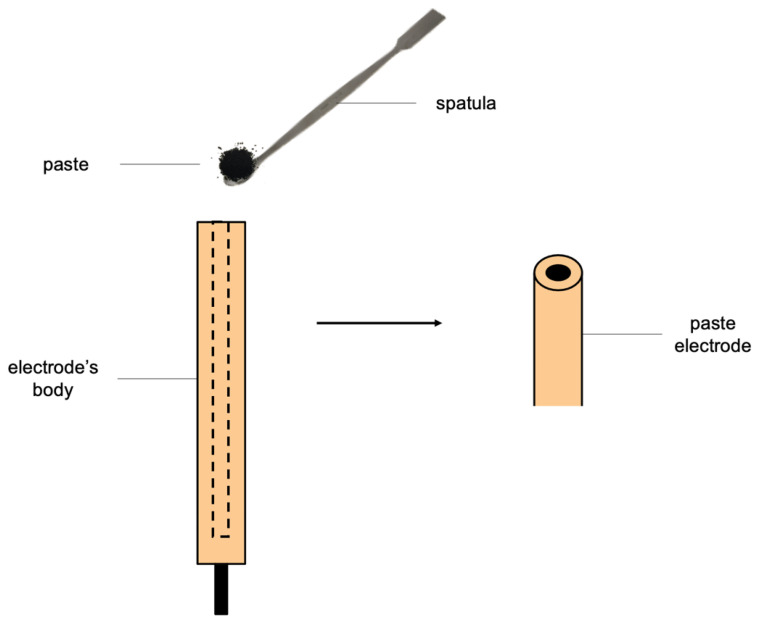
The schematic representation of the paste electrode.

The alternative construction of solid-contact electrodes with the solid-contact layer between the membrane and the electrode is the construction of a paste electrode in which the material fills the electrode corpus (as presented in Figure 3). In 1958, Adams was the first to incorporate carbon paste electrodes for analytical purposes [50]. The filling material is a paste of components mixed with paraffin oil, which is placed in the cavity of the electrode [51,52,53,54,55]. The leveled surface of the paste acts as a modified electrode substrate. This construction allows for the reduction in the consumption of toxic organic solvents, such as dimethylformamide and tetrahydrofuran, which are commonly used for the preparation of the solid-contact layer solution. The reduction in the use of solvents, ease of preparation, and the simplicity of construction are the main advantages of paste electrodes. In addition, paste electrodes are reusable because, after surface renewal, the electrode can be repeatedly covered with an ion-selective membrane.

Aside from the paste electrode, the construction of the solid-contact electrode can be further simplified by removing the ion-selective membrane and using metal oxide nanoparticles as the sensing layer. This approach can be applied to pH sensors, as some metal oxides are sensitive to hydrogen ions [49].

**Figure 4:**
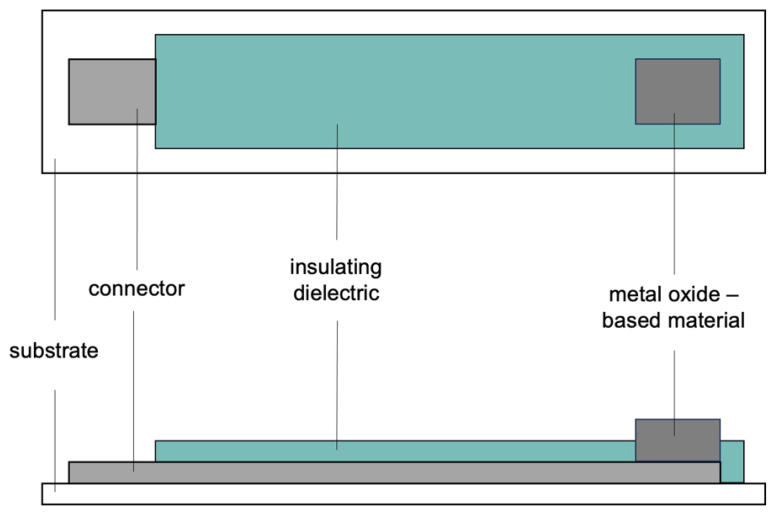
The schematic representation of the screen-printed electrode.

Various methods are used to manufacture pH sensors from metal oxides. Manufacturing methods include thermal or anodic oxidation of the metal, decomposition of a metal salt onto a back contact, and pressing of oxide pellets to hold the oxide in an inert matrix. MO_x_-based sensors can be manufactured using screen printing [56,57,58,59], sputtering [60,61], sol-gel [62,63], and electrodeposition [64,65] methods. One of the most common methods is to use thick-film screen-printing techniques [66,67,68,69]. Planar technologies are employed for developing solid-state sensors of low cost, small size, and high reproducibility. Screen printing is strongly recommended as a simple and fast method for the mass production of disposable electrochemical sensors. pH sensors were one of the first types of potentiometric sensors investigated for implementation using thick-film technology. The primary studies focused on the attempt to mimic the structure of a conventional glass pH electrode in a thick-film form [70]. The screen-printing technique allows one to produce microelectrodes by casting a small amount of material with a controlled thickness [71]. An exemplary and schematic construction of a screen-printed metal oxide-based electrode is presented in Figure 4.

## 3. Metal Oxides

This review focuses on the application of metal oxides in potentiometric sensors. In this chapter, we provide the characterization of metal oxides in the context of designing several types of sensors.

### 3.1. Characterization of Metal Oxides as Electrode Materials

One of the most known constructions of a potentiometric sensor is the solid-contact electrode. When choosing the material for solid-contact layers in potentiometric sensors, some key aspects must be taken into careful consideration.

Firstly, the material must enable an efficient ion-electron exchange process at the electrode | ion-selective membrane interface. Those processes are associated with either high redox capacitance or high double-layer capacitance. Redox capacitance is associated with the electron and ionic conductivity of materials in which exchange processes occur through reversible redox reactions, while double-layer capacitance surface development results from the nanometric size of particles or pores in the material structure.

Due to the nanometric size of its particles, nanostructured metal oxides are characterized by a high surface area. Besides its favorable morphology, some metal oxide nanomaterials such as ruthenium or iridium dioxide exhibit excellent redox properties. One of the mechanisms proposed by Fog et al. in [49] is that in contact with hydrogen ions, the oxide undergoes a redox reaction in which ions and protons are exchanged. This mechanism decides the ability of the oxide to transduce both ions and electrons through the layer, which simplifies the processes on the interface between the ion-selective membrane and the electronic conductor. The combination of those two properties—high surface area and high redox capacitance [4]—in one material makes it a great choice for the solid-contact layer.

In addition to the basic criterion of the presence of mixed ion-electron conductivity and high electrical capacitance, materials for solid-contact layers must meet a number of other requirements, including high hydrophobicity, chemical stability of the material, and the absence of side reactions occurring during the electron-ion exchange process [22,27,72,73].

The wettability of the solid-contact material is of particular importance in the context of the formation of an undesirable aqueous layer between the ion-selective membrane and the electron conductor. In the literature, hydrophilic materials (characterized by low values of the wetting angle below 90°) introduced into the electrode design did not prevent the formation of a water layer, unlike hydrophobic materials (with wetting angles above 90°), which have been repeatedly proven to protect sensors from the unwanted water layer and susceptibility to drop off of the ion-selective membrane [35,74,75]. Metal oxides, especially hydrous forms of oxides, are characterized by low wetting angle values and are defined as a hydrophilic material. Besides this property, even after prolonged use and conditioning of the electrodes in aqueous solutions, an aqueous layer did not form. This may be due to a specific reaction occurring on the surface of the ruthenium oxide layer [49] and/or large surface development that conditions the solid-contact layer to adhere strongly to the membrane, preventing it from peeling off the electrode surface.

The opposite requirements concerning wettability relate to electrodes without an ion-selective membrane. In this construction of an electrode, the metal oxide nanoparticle layer contacts the analyzed fluid and must be easily wettable by aqueous solutions. Taking this into account, hydrophilic metal oxides with contact angles below 30 degrees are perfect for the sensing layer in potentiometric sensors for pH measurement.

In the context of the design of paste electrodes, the material used as a paste should meet certain requirements. These include providing a quick and reversible transition from ionic to electronic conductivity and having a nonpolarizable surface between the paste and the high-capacity red-ox or double-layer ISM. In addition, the paste should be hydrophobic and characterized by low water absorption, which prevents the formation of a water layer. Moreover, the materials for the paste should be readily available and non-toxic. Similar to the case of solid-contact electrodes, metal oxides were proven to be great materials for paste electrode modification and production [76].

In addition, metal oxides exhibit a combination of unique properties, such as high thermal and chemical stability and low resistivity [11].

### 3.2. The Action Mechanism of Metal Oxides

The action mechanism is described based on the behavior of ruthenium oxide after contacting the electrolyte. According to the reaction mechanism proposed by Fog and Buck [49], ruthenium oxide in contact with hydrogen ions undergoes redox reactions, in which both ions and charged elementary parts are exchanged. The action mechanism of metal oxides is based on the redox reactions. These processes are initiated by electrons (e−) and protons (H+) introduced to the surface of the dioxide from the electrolyte, as presented in the following reaction [77]:

RuO_2_ + xH + xe = RuO_2_ − x(OH)_x_, where 0 ≤ x ≤ 2

This mechanism is used indirectly when designing sensors for pH determination with metal oxides acting both as a sensing layer and as a solid-contact layer.

In the literature [78], it was noted that the insertion of other cations, for example, potassium ions, into the porous dioxide layer also participates in the mentioned mechanism. As presented by Wen et al. [79], monovalent cations may act as hydrogen ions in the presented reaction and, as protons, lead to faradaic reactions. This feature was used when designing potentiometric sensors with an ion-selective membrane based on metal oxides for potassium determination [80].

**Figure 5:**
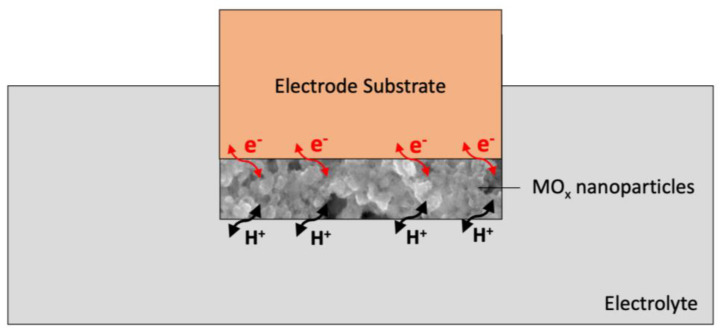
Mechanism of ion-electron exchange at phase boundaries using a metal oxide (MO_x_) layer.

The schematic representation of the charge storage mechanism that occurs via the proton-electron transfer for the metal oxide (MO_x_) layer is presented in Figure 5.

## 4. Metal Oxide Nanoparticles as a Sensing Material

Metal oxides as sensing materials are mostly used in pH sensors. Historically, the use of metal oxides in potentiometric sensors began in the 1980s when Fog and Buck introduced them for the first time as a sensing material for pH detection [49]. Some of the metal oxides were successfully applied as pH sensor materials and showed a near-Nernstian response in the pH range of 2–12. This research was followed by many publications by scientists searching for a replacement for the glass-membrane electrode.

Among the oxides used for the fabrication of pH sensors, the most commonly known and used are ruthenium oxide RuO_2_ and iridium oxide IrO_2_, characterized by remarkable sensitivity to hydrogen ions and high accuracy. Furthermore, we can distinguish tantalum(V) oxide Ta_2_O_5_, titanium dioxide TiO_2_, tin(IV) oxide SnO_2_, cerium dioxide CeO_2_, tungsten trioxide WO_3_, lead dioxide PbO_2_, and mixed oxides: Bi_2_O_3_-Nb_2_O_5_, IrO_2_-TiO_2_, and RuO_2_-TiO_2_ for pH sensor applications. In addition to this, some work has been conducted in the area of metal/MO_x_-based pH sensors such as those based on antimony and bismuth. In these couples, the redox equilibrium between the metal/MO_x_ phases (e.g., Sb-Sb_2_O_3_) is attributed to the origin of their pH sensitivity [81,82,83]. The sensitivity of the MO_x_ pH sensor significantly depends on the type of material composition and the deposition method since both factors can influence the microstructure, porosity, surface homogeneity, and crystalline structure of the material. This leads to variation in the sensitivity of the electrodes. The sensitivity of the pH sensors is expressed in terms of the Nernstian response (the sensor is expected to show 59.14 mV/pH at 25 °C derived from the Nernst equation). We investigated the sensitivity of different MO_x_ including IrO_2_ [84,85], RuO_2_ [58,60,86,87,88,89], TiO_2_ [86,89,90], SnO_2_ [88,91], Ta_2_O_5_ [58,61], WO_3_ [92,93], CeO_2_ [94], PbO_2_ [95], MnO_2_ [96], and CoO_2_ [97] and compared them with the theoretical sensitivity. Table 1 presents the comparison of analytical parameters of MO_x_-based sensors such as pH measurement range, slope of calibration curve, and time of response.

## 5. Metal Oxide Nanoparticles as Solid-Contact Layers

One of the observed trends in the development of potentiometric sensors is the search for new functional materials for the construction of solid-contact electrodes. These electrodes are characterized by sandwich-like construction with a solid-contact layer between the substrate electrode and the ion-selective membrane. The properties of the solid-contact layer directly translate into the properties of the sensors, allowing them to achieve lower and lower limits of determination, higher potential stability, and lower response times.

As already mentioned, one of the trends in modern potentiometry is the search for new materials for solid-contact layers, which, because of their properties, will improve the analytical and performance of solid-contact ion-selective electrodes. Since the 1990s, when conductive polymers were first introduced as a solid-contact layer, up to the present day, various types of materials have been introduced into electrode design with varying results [4,13].

A group of metal oxides can be classified as high redox capacity and high double-layer capacity materials, as they exhibit both high redox sensitivity and high surface area [49]. The implementation of metal oxide macro/nanomaterials as solid-contact layers, to support the ion-to-electron transduction processes, relies on two strategies: High specific surface area and high redox activity. Some of the tested metal oxides also appeared to meet all the other requirements for solid-contact layers; hence, they were considered promising materials for solid-contact electrode design.

The mechanism of the selective response to hydrogen ions, described and applied by Fog and Buck [49], was later implemented by many research groups in the design of ion-selective electrodes with solid-contact layers based on metal oxide.

The first implementation of metal oxide in membrane-based potentiometric sensors dates back to 2013 when the Khun group introduced ZnO nanorods as solid-contact material for Cr^2+^-ISEs and ZnO nanotubes in I-ISEs [98]. The hollow structures of ZnO nanotubes can provide a larger surface area than ZnO nanorods. Later, the same group presented the use of CuO nanoflowers in Cd^2+^-ISEs [99]. These electrodes exhibit a very advantageous LOD (level of detection) at the nanomolar level. In other studies, the Qin group proposed MoO_2_ microspheres as an ion-to-electron transducer (2017) [100]. Compared to the coated-disc electrodes, the introduction of MoO_2_ microspheres improves the sensor performance to some extent as a result of the enhanced double-layer capacitance. In 2019, Yang et al. manufactured solid-contact K^+^-ISEs based on MnO_2_ nanosheets for the determination of K^+^ in the blood [101]. The MnO_2_ nanosheets turned out to have a wrinkled morphology, thereby preventing their dense accumulation and maintaining the advantage of a large surface area, which is advantageous for achieving a large double-layer capacitance. Also in 2019, the Paczosa-Bator group introduced hydrous RuO_2_ nanoparticles into solid-contact H^+^—and K^+^-ISEs as ion-to-electron transducers. Remarkably, these RuO_2_ nanoparticle-based sensors exhibit a large redox capacitance at the millifarad level. Other oxides introduced by the Paczosa-Bator group in the construction of ion-selective electrodes were hydrous IrO_2_ (2021) [102], and a year later, hydrous CeO_2_ (2022) [103]. In 2022, Wardak’s group presented a paper on the use of ZnO, CuO, and Fe_2_O_3_ in potassium-sensitive electrodes [104].

### Metal Oxide Nanoparticles-Based Hybrid Materials as Solid-Contact Layers

Metal oxides were also used as components of hybrid materials with remarkable success. In 2019, Zeng et al. introduced TiO_2_-PANI material into the Pb^2+^- selective electrodes. This material possessed a large specific capacitance, thereby effectively promoting ion-to-electron transduction and enhancing the potential response stability of electrodes [105].

Later, the Paczosa-Bator group presented composite materials containing RuO_2_ nanoparticles and conducting polymers POT (RuO_2_–POT) [106] and PEDOT:PSS [107] and composite material containing ruthenium dioxide and carbon nanomaterials [108]. All the mentioned hybrids were applied as solid-contact layers in K^+^- selective electrodes.

The RuO_2_–POT composite exhibits superhydrophobic properties (a contact angle of 149°). The results of electrochemical techniques demonstrate that these solid-contact electrodes possess a large capacitance of approximately 1.2 mF, which is much larger than that of the coated-disc electrode. Compared to single-component counterparts, K^+^-ISE based on the RuO_2_-POT composite exhibits a shorter equilibration time and better potential stability and reproducibility.

The RuO_2_-PEDOT:PSS composite allowed the acquisition of electrodes with an electrical capacitance of as much as 7 mF. It is worth mentioning that the value of 7.2 mF is one of the highest values presented in the literature for this type of electrode.

The addition of graphene, the representative of the carbon materials group, to ruthenium dioxide allowed the capacitance to increase to 2.6 mF, which is 2.5 times higher compared to the oxide alone.

In 2021, Su et al. designed electrodes with MoS_2_-Fe_3_O_4_ as an ion-electron transduction layer for the determination of serum potassium [109]. These sensors present high performance in potential reproducibility and long-term stability.

In 2022, Lenar et al. presented a composite material based on iridium dioxide, creating a hybrid material of not two but three different components. The layer of IrO_2_-POT-MWCNT turned out to be superhydrophobic with a contact angle of 178°, which resulted in great stability in potentiometric response and long lifetime. This layer was introduced in K^+^ [110] and H^+^ [111] selective electrodes.

A year later, the group of Wardak presented the CuO-MWCNT material, applied as a solid-contact layer in Cu^2+^-selective electrodes [112]. These electrodes were characterized by a low detection limit (1.5 × 10^–8^ mol L^–1^) and very good potential stability.

## 6. Metal Oxides Nanoparticles in Paste Electrodes

Both metal oxides and their composites were used as a component in the paste electrodes. Niemiec et al. designed carbon black paste electrodes modified with poly(3-octylthiophene-2,5-diyl) and transition metal oxides: Ruthenium and iridium dioxide [77].

In this research, electrodes with carbon paste based on carbon black and modified with hydrated transition metal oxides, namely hydrous ruthenium dioxide and hydrous iridium dioxide, were presented. The paste with hydrous ruthenium dioxide, characterized by the best capacitance (470 μF), was extra modified by poly(3-octylthiophene-2,5-diyl). This modification results in a PVC-based electrode with the highest capacitance (130 μF) and, consequently, the lowest drift of the potentiometric response. The developed electrodes can be used to determine nitrate ions in liquid samples and soil samples [77].

## 7. Comparative Study

Table 2 presents a comparison between the analytical parameters of the solid-contact electrodes sensitive to various ions with the solid-contact layer based on the nanoparticles of the metal oxide.

Parameters were selected according to their importance and influence on the sensor performance. For each sensor presented in the literature, the slope of the calibration curve, linear range, stability of the potentiometric response described by the potential drift, and electrical capacitance parameters were compared. To provide the reader with comparable data, the potential drift values in the table refer to the potential drift parameter determined during the long-term potentiometric measurement. Since there are only a few methods that allow the determination of the electrical capacity of an electrode and may vary depending on the electrochemical technique used, the electrical capacitance parameter provided in the table is the parameter obtained from the chronopotentiometric measurement.

## 8. Summary

This review reveals that metal oxides are widely used for the design of potentiometric sensors. In the literature, many reports can be found on the usage of metal oxide nanoparticles in sensors of various constructions: Solid-contact electrodes, paste electrodes, and screen-printed electrodes. Because of their unique properties, metal oxides can be applied both as sensing layers in pH sensors and as solid-contact layers in sensors with a polymeric membrane. Metal oxide nanoparticles were incorporated into H^+^-selective thin-film screen-printed electrodes as the sensing material and in solid-contact and paste electrodes with a polymeric membrane for the determination of various ions. The presence of a membrane allows for designing sensors sensitive to various ions, not only H^+^ ions. The comparative study performed within the scope of this review displays that the presence of metal oxides in the sensor construction allows for obtaining robust analytical tools with a short response time and great potential stability. This is guaranteed by the high electrical capacity of the metal oxides, thanks to their remarkable redox properties and the nanometric size of the particles. Because of the great analytical parameters, low cost of the equipment and single analysis, and endless modification possibilities, potentiometry with the use of metal oxide-based electrodes can be considered a forward-looking method for future problems.

The challenges in the field of potentiometric sensors that still need to be faced concern lowering the detection limit for trace-level analysis and improving the stability of potentiometric response in order to eliminate the need for calibration in the future.

Based on the many literature reports, it can be stated that the application of metal oxides is promising in the context of designing potentiometric sensors, and their introduction into sensor construction may be considered a breakthrough in this field. Because of their high electrical capacity, metal oxides improve the stability of the potentiometric response of sensors and allow for a reduction in the number of necessary calibrations. As a consequence, this may lead to the creation of calibration-free sensors, which is the most sought-after feature in research on electrochemical sensors.

The area of metal oxides will possibly continue to develop towards composite materials designed with the use of substrates representing various groups of materials. This approach allows one to combine the best features into one material of new, unique properties and to modify metal oxides by enhancing their electrical capacity or hydrophobicity.

The application of ion-selective membrane over the metal oxide layer in solid-contact electrodes allows for the detection of various ions and there is a great possibility to extend their detection capabilities beyond common ions. The miniaturization and creation of flexible versions of potentiometric sensors achieved by thick-film technologies can broaden the application fields of metal oxide screen-printed electrodes, such as in vivo biomedical measurements and wearable point-of-care analysis.

## Figures and Tables

**Table 1:** pH sensors based on metal oxides—metal oxides as sensing materials.

Material	pH Range	Slope [mV/pH]	Response Time	Reference
RuO_2_	1–13	55.64	-	[60]
RuO_2_-TiO_2_	2–12	56.03	-	[90]
RuO_2_-Ni	1.5–12.5	52	-	[86]
RuO_2_-NT	2–12	55	<40 s	[87]
RuO_2_-Ta_2_O_5_	2–12	56	<8 s	[58]
RuO_2_-SnO_2_	2–12	56.5	<5 s	[88]
IrO_2_	2–10	63.5	~1 min	[84]
IrO_2_-TiO_2_	1–13	59.1	120 s	[85]
Ta_2_O_5_	2–12	58–59	<0.3 s	[61]
SnO_2_	2–12	58.1	-	[91]
TiO_2_	1–11	58.73	-	[89]
WO_3_	1–7	44.85	-	[92]
WO_3_-NT	2–12	41	<90 s	[93]
CeO_2_	7.2–10.8	38	-	[94]
PbO_2_	1.5–12.5	64.82	<1 s	[95]
MnO_2_	2–12	78.3	few seconds	[96]
CoO_2_	1–12	54.9	<1 min	[97]

List of abbreviations: NT—nanotubes, IrO_2_—iridium dioxide, RuO_2_—ruthenium dioxide, MnO_2—_manganium dioxide, CeO_2_—cerium dioxide, TiO_2_—titanium dioxide, Ta_2_O_5_—tantalum(V) oxide, SnO_2_—tin(IV) oxide_,_ WO_3_—tungsten trioxide, PbO_2_—lead dioxide, CoO_2_—cobalt(II) oxide.

**Table 2:** The comparison of analytical parameters of solid-contact electrodes with solid-contact layer based on metal oxides nanoparticles—metal oxides as solid-contact layers.

Solid-Contact	Ion	Slope, pX/dec	Linear Range, M	Potential Drift *, mV/h	Electrical Capacity **, μF	Reference
RuO_2_	H^+^	59.31	10^−12^–10^−2^	0.15	1120	[113]
IrO_2_	H^+^	54.12	10^−11^–10^−2^	0.1	66	[111]
IrO_2_-NT	H^+^	54.40	10^−11^–10^−2^	0.077	174	[111]
IrO_2_-NT-POT	H^+^	57.18	10^−11.5^–10^−2^	0.036	387	[111]
MoO_2_ microspheres	K^+^	55.0	10^−3^–10^−5^	0.012	86	[100]
MnO_2_ nanosheets	K^+^	51.85	10^−2^–10^−5^	-	29	[96]
CuO	K^+^	56.68	10^−1^–10^−5^	0.54	0.104	[104]
ZnO	K^+^	56.18	10^−1^–10^−5^	0.16	0.026	[104]
Fe_2_O_3_	K^+^	55.11	10^−1^–10^−5^	1.40	0.010	[104]
RuO_2_	K^+^	57.37	10^−1^–10^−6^	0.0015	1233	[114]
RuO_2_-POT	K^+^	58.64	10^−6^–10^−1^	0.028	1170	[106]
RuO_2_- PEDOT:PSS	K^+^	58.93	10^−6^–10^−1^	0.077	7200	[107]
RuO_2_-GR	K^+^	58.95	10^−6^–10^−1^	-	2600	[108]
RuO_2_-NT	K^+^	58.25	10^−6^–10^−1^	-	1050	[108]
RuO_2_-CB	K^+^	58.03	10^−6^–10^−1^	-	1080	[108]
IrO_2_	K^+^	59.29	10^−6^–10^−1^	0.063	920	[102]
IrO_2_-POT-NT	K^+^	57.33	10^−6^–10^−1^	0.043	1500	[110]
CeO_2_	K^+^	55.32	10^−5^–10^−1^	0.086	9	[103]
CeO_2_-NT	K^+^	58.90	10^−6^–10^−1^	0.095	610	[103]
CeO_2_-POT	K^+^	58.21	10^−6^–10^−1^	0.24	96	[103]
ZnO nanorods	Cr^2+^	28.65	10^−6^–10^−1^	-	-	[98]
ZnO nanotubes	I^−^	62	10^−6^–10^−1^	-	-	[98]
CuO nanoflowers	Cd^2+^	29.3	10^−9^–10^−1^	-	-	[99]
TiO_2_-PANI	Pb^2+^	29	10^−9^–10^−1^	-	8.1	[105]
MoS_2_-Fe_3_O_4_	K^+^	55.2	10^−5^–10^−2^	-	350	[109]
CuO-NT	Cu^2+^	30.1	5 × 10^−8^–3 × 10^−2^	-	-	[112]
CB-IrO_2_ (paste)	NO_3_^−^	−57.2	10^−5^–10^−1^	0.11	86	[76]
CB-RuO_2_ (paste)	NO_3_^−^	−57.2	10^−5^–10^−1^	0.11	98	[76]
CB-RuO_2_-POT (paste)	NO_3_^−^	−56.9	10^−5^–10^−1^	0.02	130	[76]

* Potential drift parameter determined during the long-term potentiometric measurement. ** Electrical capacitance parameter obtained from the chronopotentiometric measurement. List of abbreviations: POT—poly(3-octylthiophene-2,5-diyl, NT—nanotubes, IrO_2_—iridium dioxide, RuO_2_—ruthenium dioxide, MoO_2_—molybdenum dioxide, MnO_2—_manganium dioxide, CuO—copper oxide, ZnO—zinc oxide, Fe_2_O_3_—iron(III) oxide, PEDOT:PSS—poly(3,4-ethylenedioxythiophene) polystyrene sulfonate, GR—graphene, CB—carbon black, CeO_2_—cerium dioxide, PANI—polyaniline, TiO_2_—titanium dioxide, Fe_3_O_4_—iron(II,III) oxide, MoS_2_—molybdenum disulfide.

## Data Availability

Data sharing is not applicable to this article.
